# Cleft Palate in Apert Syndrome

**DOI:** 10.3390/jdb10030033

**Published:** 2022-08-11

**Authors:** Delayna Willie, Greg Holmes, Ethylin Wang Jabs, Meng Wu

**Affiliations:** Department of Genetics and Genomic Sciences, Icahn School of Medicine at Mount Sinai, New York, NY 10029, USA

**Keywords:** Apert syndrome, cleft palate, FGF, FGFR2, high-arched palate, palatogenesis, pseudo-cleft palate, uvula

## Abstract

Apert syndrome is a rare genetic disorder characterized by craniosynostosis, midface retrusion, and limb anomalies. Cleft palate occurs in a subset of Apert syndrome patients. Although the genetic causes underlying Apert syndrome have been identified, the downstream signaling pathways and cellular mechanisms responsible for cleft palate are still elusive. To find clues for the pathogenic mechanisms of palatal defects in Apert syndrome, we review the clinical characteristics of the palate in cases of Apert syndrome, the palatal phenotypes in mouse models, and the potential signaling mechanisms involved in palatal defects. In Apert syndrome patients, cleft of the soft palate is more frequent than of the hard palate. The length of the hard palate is decreased. Cleft palate is associated most commonly with the S252W variant of FGFR2. In addition to cleft palate, high-arched palate, lateral palatal swelling, or bifid uvula are common in Apert syndrome patients. Mouse models of Apert syndrome display palatal defects, providing valuable tools to understand the underlying mechanisms. The mutations in FGFR2 causing Apert syndrome may change a signaling network in epithelial–mesenchymal interactions during palatogenesis. Understanding the pathogenic mechanisms of palatal defects in Apert syndrome may shed light on potential novel therapeutic solutions.

## 1. Introduction

Orofacial clefts (OFCs) are the most common craniofacial birth defects [1]. OFCs cause health issues and complications early in life such as feeding problems and ear infections, increasing morbidity and mortality risks [2]. Treatments including surgery, speech therapy, and dental management usually are required. In addition, OFCs cause significant psychological and socioeconomic difficulties for both the patient and the family, and the effects may extend through adulthood [3].

OFCs can be classified as cleft lip with or without cleft palate (CL/P) and cleft palate only (CPO) [1]. The prevalence is 3.1 per 10,000 live births for cleft lip only, 5.6 per 10,000 for cleft lip with cleft palate, and 5.9 per 10,000 for cleft palate only [4]. CPO is a multifactorial disorder caused by both genetic and environmental factors [5,6]. CPO can be categorized into non-syndromic (isolated) and syndromic CPO [5]: non-syndromic CPO is an isolated condition unassociated with any other recognizable anomalies; syndromic CPO is associated with abnormalities in addition to the cleft or with a syndrome with a known genetic etiology [7].

Apert syndrome (MIM #101200) is a congenital disorder characterized by clinical features including multisuture craniosynostosis, midface retrusion, and syndactyly of the hands and feet [8]. It occurs in about 1:80,000 to 1:160,000 live births [9,10,11]. The genetic causes of Apert syndrome are variants affecting the fibroblast growth factor receptor 2 (*FGFR2*) gene. The human *FGFR2* gene is located on chromosome 10q26 and encodes a receptor tyrosine kinase. FGFR2 consists of an extracellular portion composed of three immunoglobulin-like domains (IgI, IgII, and IgIII) responsible for extracellular ligand binding, a transmembrane region, and an intracellular tyrosine kinase domain [12,13,14]. More than 98% of Apert syndrome cases are caused by two amino acid substitutions, Ser252Trp (S252W) and Pro253Arg (P253R), in the linker region between the second and third extracellular Ig domains [15,16]. Approximately 67% of Apert syndrome cases have the S252W variant, while P253R accounts for 32% of cases [15,16,17]. Other rare variants include Ser252Phe (S252F) [17,18,19,20], Ser137Trp (S137W) [21], Alu-element insertions in *FGFR2* [22], and a deletion between *FGFR2* exons IIIb and IIIc creating a chimeric IIIb/IIIc exon [23]. These are all gain-of-function *FGFR2* mutations [24,25].

Palatogenesis involves initiation, growth, morphogenesis, and fusion of the primary and secondary palatal shelves [26]. The primary palate is derived from the medial nasal processes. In humans, the primary palate begins to form during the fifth week of gestation, and fusion of the primary palate is complete by the sixth week [27]. Secondary palatal shelves develop as outgrowths from maxillary prominences. The secondary palate fuses anteriorly with the primary palate and anterodorsally with the nasal septum to form the roof of the oral cavity [28]. Proper growth of the secondary palate is important for its functions in closing off the airways, mastication, swallowing, and speech. In humans, secondary palatal shelves begin to develop around gestation week six. The palatal shelves develop from the inner part of the maxillary processes and extend along the lateral walls of the oropharynx [29]. The bilateral palatal shelves first grow vertically to allow for proper development of the tongue and then shift horizontally to lay above the tongue. The palatal shelves grow towards one another and eventually fuse together to create the palate [28]. Differential patterning and cellular heterogeneity along the anterior/posterior (AP) axis and along the mediolateral axis are important in palatogenesis. The anterior palatal shelf gives rise to the hard palate and the posterior portion becomes the soft palate [28]. Palatogenesis has been reviewed in more detail previously [26,28,29,30]. Genetic and environmental perturbations of the process may cause cleft palate. The objectives of this review are to clarify the palatal phenotypes in patients with Apert syndrome as well as in mouse models of Apert syndrome, and discuss the signaling pathways downstream of FGFR2 in palatogenesis to further understand the pathogenesis of cleft palate in Apert syndrome.

## 2. Materials and Methods

To review clinical characteristics of the palate in patients with Apert syndrome, PubMed was searched for relevant articles. The keywords “Apert syndrome”, “acrocephalosyndactyly”, “palate”, “palatal”, and “bifid uvula” were combined with the Boolean operators “AND” and “OR”. The search strategy (“Apert syndrome” [All Fields] OR “acrocephalosyndactyly” [All Fields]) AND (“palate” [All Fields] OR “palatal” [All Fields] OR “bifid uvula” [All Fields]) was used, resulting in 192 papers. Two researchers (DW and MW) examined these papers and excluded those for cases of Apert syndrome without palatal phenotypes, non-Apert syndrome studies, or papers written in non-English languages. The following information was retrieved from the remaining, relevant papers (*n* = 41): from cohort studies, we extracted information including sample sizes, numbers of patients with palatal phenotypes, genotypes, and palatal phenotypes; from case reports, we extracted gender, palatal phenotypes, and genotypes.

## 3. Clinical Characteristics of the Palate in Apert Syndrome

**Figure 1:**
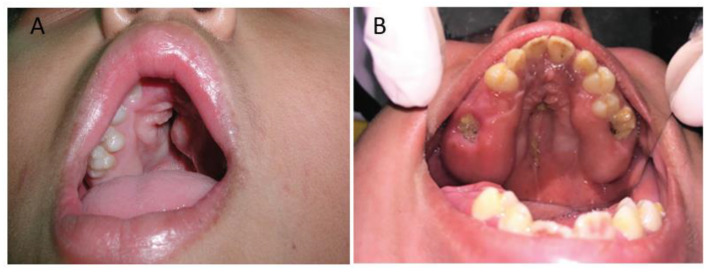
Cleft palate and high-arched palate in Apert syndrome. (**A**) Cleft palate in a patient with Apert syndrome (adapted from [48] and used with permission from Dermatology Online Journal: https://escholarship.org/uc/doj). (**B**) High-arched palate and gingival enlargement in a patient with Apert syndrome (adapted from [70]).

Apert syndrome patients present with anomalies of the palate, which may or may not include cleft palate (Table 1 and Table 2). Cleft palate (Figure 1A) is present in a subset of affected individuals with Apert syndrome [8] and the frequency is higher than control subjects [31]. However, the incidence of cleft palate in patients with Apert syndrome varies between different studies. Kreiborg and Cohen reported that in a study of 119 patients with Apert syndrome, 75% of the patients had a cleft of the soft palate or bifid uvula [32]. In a group of seven Japanese patients with Apert syndrome, Kobayashi et al. reported two patients with a cleft of the soft palate (28.6%) and one with a cleft of the hard palate (14.3%) [33]. In 17 cases of Apert syndrome reported by Arroyo Carrera et al., cleft palate was identified in 23.5% of patients [34]. In a retrospective study in Brazil, only 1 of 23 (4%) Apert syndrome patients presented with a true cleft palate [35].

Cohort studies have shown that a high-arched palate (also described as “high palate”, “pseudo-cleft”, “vaulted palate”, or “Byzantine arch-shaped palate”) with lateral palatal swelling is the most common palatal anomaly in patients with Apert syndrome (Table 1), usually present in more than 90% of the patients [33,35,36,37,39,43]. It also is present frequently in case reports of Apert syndrome (Table 2). Patients with narrow, high-arched palates (Figure 1B) and/or gingival swellings [33,35,37,39], may lead to a misdiagnosis of cleft palate in early studies [37].

Apert syndrome has a high frequency of soft palate cleft or bifid uvula [32]. Clefts of the soft palate are more frequent than of the hard palate in patients with Apert syndrome [33,35]. Bifid uvula, which is a split uvula, is often considered as a marker for submucous cleft palate (SMCP) [74,75]. SMCP is a subgroup of cleft palate resulting from insufficient medial fusion of the muscles of the soft palate during palatogenesis [75]. A submucous cleft palate may appear to be structurally intact, but other defects may be present, including a bony notch in the hard palate, a bluish line at the midline of the soft palate (zona pellucida), and a bifid uvula [27,76].

In addition to the clefts, decreased length of the hard palate has been observed in patients with Apert syndrome [38,77]. This may indicate defects in palatal bone formation caused by the pathogenic FGFR2 variant, or it could be secondary to midface hypoplasia, which is one of the features of Apert syndrome.

Genotype–phenotype correlations in Apert syndrome have been studied. Although these correlations are variable, cleft palate is associated more commonly with the S252W variant than P253R in multiple studies comparing subgroups defined by these two variants in FGFR2 [16,17,78,79,80,81]. Cleft palate is present in approximately 60% of patients with the S252W variant and 15% of patients with the P253R variant (Table 3), suggesting this genotype–phenotype correlation in Apert syndrome.

## 4. Palatal Phenotypes in Mouse Models of Apert Syndrome

Palatal structure and development are very similar between humans and mice [28]. Many genes involved in cleft palate have been identified using genetically modified mouse models, and the morphological and molecular analyses of these models have contributed to our understanding of the mechanisms of palatogenesis and pathogenesis in disease [26,28,29,30].

A variety of mouse models of Apert syndrome have been created. These models display many of the phenotypes found in human Apert syndrome [82]. Two independent mouse models of both FGFR2 S252W [83,84] and FGFR2 P253R [85,86] have been generated and characterized. In addition to these models, another was created by deletion of *Fgfr2* exon IIIc [87]. FGFR2 has two major isoforms. One is expressed in epithelia and contains exon IIIb, while the other is expressed in mesenchyme and contains exon IIIc [88,89]. Deletion of exon IIIc caused a splicing switch that resulted in ectopic expression of the epithelial IIIb isoform within mesenchyme and neural tissue. This represents a model of the Alu splice-switch mutations and the phenotypes parallel some of those found in Apert and Pfeiffer syndrome patients [22,87].

**Figure 2:**
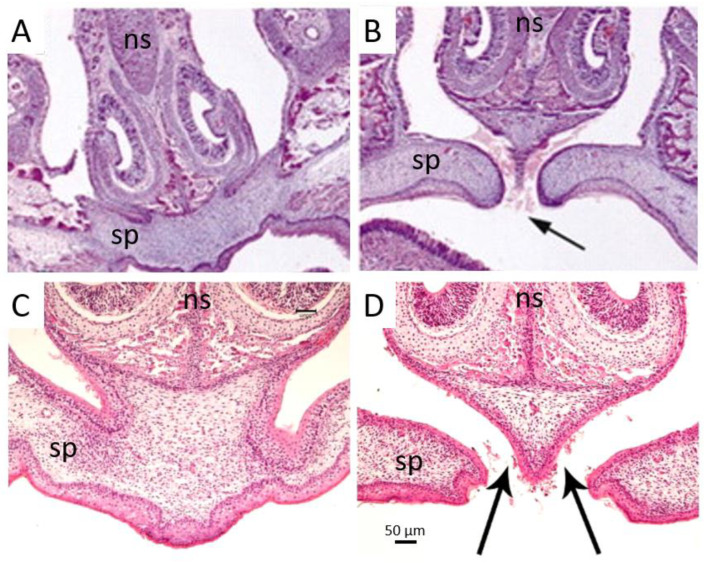
The palatal phenotypes of FGFR2 S252W and P253R Apert mouse models. (**A**,**B**) Incomplete closure of the anterior *Fgfr2^+/2S252W^* secondary palate at P1 (**B**) compared to WT (**A**) (adapted from [84] and used with permission from Development: dev.biologists.org). (**C**,**D**) Incomplete closure of the anterior *Fgfr2^+/P253R^* secondary palate at P0 (**D**) compared to WT (**C**) (adapted from [85]). ns, nasal septum; sp, secondary palate. Arrows indicate incomplete fusion.

In both mouse models of FGFR2 S252W, malformation of the palate was discovered [84,90] (Table 4). In *Fgfr2^+/S252W^* mice, there was incomplete fusion of the primary and secondary palates at the midline incisive foramina, evident by P0 (Figure 2A,B). The structures of the face and palate were 40–50% smaller in the mutant mouse compared to control littermates [84], which is similar to the shorter palate in patients with Apert syndrome [77].

Similar to *Fgfr2^+/S252W^* mice, *Fgfr2^+/P253R^* mice exhibited incomplete fusion at the junction of the primary and secondary palates at the incisive foramina, evident from E15.5 [85] (Figure 2C,D). The developing palates of *Fgfr2^+/P253R^* mice were also shorter than control littermates [85].

Martínez-Abadías et al. analyzed high-resolution μCT images of newborn mouse skulls to precisely quantify distinct palatal phenotypes of *Fgfr2^+/S252W^* and *Fgfr2^+/P253R^* mice [91]. *Fgfr2^+/S252W^* mice displayed relatively more severe palatal dysmorphology, with contracted and more separated palatal osteogenic fronts, a greater tendency to fuse the maxillary–palatine sutures, and aberrant development of the inter-premaxillary suture. These palatal defects are associated with suture-specific patterns of abnormal cellular proliferation, differentiation, and apoptosis [91]. These results reveal palatal phenotypes in addition to clefts, and the important role of FGFR2 in the formation of bones and sutures that shape palatal morphogenesis.

To achieve conditional expression of mutant *Fgfr2* in mouse models, researchers have used tissue-specific or inducible Cre recombinase mouse lines. This can avoid the typical lethality of Apert syndrome mutations in mice to allow the study of postnatal aspects of the condition, and also provides insights into tissue-specific contributions to the phenotype. Holmes and Basilico used global EIIA-Cre, neural crest-specific *Wnt1*-Cre, and mesoderm-specific *Mesp1*-Cre to induce expression of FGFR2 S252W [90]. Similar to Wang et al.’s results [84], *EIIA-Cre*;*Fgfr2^Neo-S252W/+^* pups at P0 exhibited failure of fusion between the secondary and primary palates at the incisive foramina [90]. When the mutation was restricted to the neural crest-derived mesenchyme from which the palatal mesenchyme is derived in *Wnt1-Cre*;*Fgfr2^Neo-S252W/+^* mice, or to mesoderm-derived mesenchyme in *Mesp1-Cre;Fgfr2^Neo-S252W/+^* mice, palatal fusion was complete (Table 4). These results suggest that the fusion defect is due to defects in the epithelium [90].

## 5. Molecular and Cellular Mechanisms

Palatogenesis is a dynamic process precisely regulated by multiple signaling pathways in a temporal and spatial manner. Crosstalk between pathways and reciprocal interactions between the epithelium and the mesenchyme are crucial for palatal growth and patterning [26,28]. The mutations in FGFR2 causing Apert syndrome may trigger a variety of changes at the molecular, cellular, and tissue levels to affect the morphology of the palate.

### 5.1. FGFR2 Signaling

In mice, *Fgfr2* is expressed in the entire palatal epithelium and the medial aspect of the posterior palatal mesenchyme from E13.5 to E14.5 [92]. At E15, when palatal shelf fusion initiates, the expression of *Fgfr2* remains in the nasal aspect of the confluent palatal mesenchyme and in medial edge epithelium (MEE) cells [92,93]. It is also expressed in the mesenchyme surrounding the ossifying palatine bones [92].

FGF signaling is critical during palatogenesis and participates in all stages of palate development, especially in cell proliferation of palatal shelves [93]. Canonical FGFs function as autocrine or paracrine factors. Binding specificity varies between particular ligands and receptors, and is modulated by heparan sulphate proteoglycans [94]. The expression of ligand/receptor pairs is often segregated between epithelium and mesenchyme, requiring signaling between the tissues for normal development [95]. FGFRs are transmembrane receptor tyrosine kinase proteins (RTKs), and immunoglobulin-like domains II and III and the linker region between these domains regulate ligand binding specificity [94]. The two most common missense variants in Apert syndrome, causing amino acid substitutions S252W and P253R, are located in the linker region of FGFR2 [15]. This region is highly conserved amongst the different FGFRs [13]. The S252W and P253R variants result in increased affinity for FGF ligand [96] and loss of ligand binding specificity [25]. Normally, mesenchymally expressed FGF7 and FGF10 are specific for the epithelial splice form FGFR2b, whereas FGF2, FGF4, FGF6, FGF8, and FGF9 are specific for the mesenchymal splice form FGFR2c [97,98]. Conversely, the FGFR2 S252W variant allows FGFR2c to bind and be activated by FGF7 and FGF10, and FGFR2b to be activated by FGF2, FGF6, and FGF9 [25]. These altered binding patterns therefore may disrupt FGF signaling between epithelium and mesenchyme.

FGFR2 connects to a complex intercellular signaling network. FGFR activation induces four major intracellular signaling pathways: RAS-MAPK (ERK1/2), PI3K-AKT, PLCγ-PKC, and signal transducer and activator of transcription (STAT) [94]. ERK1/2 is the main effector of FGF signaling [99,100]. Disruption of the ERK/MAPK pathway in neural crest cells of *Wnt1-Cre*;*Erk2^fl/fl^* mice causes cleft palate, malformed tongue, micrognathia and mandibular asymmetry [101], similar to the Pierre Robin complex, triad of mandibular dysmorphology, glossoptosis, and respiratory obstruction with or without cleft palate [102]. However, when *Erk2* deletion is restricted to the palatal mesenchyme in *Osr2-Cre*;*Erk2^fl/fl^* mice, cleft palate was not observed, indicating that the cleft palate in *Wnt1-Cre*;*Erk2^fl/fl^* mice is a secondary defect. Knockout of *Spry2*, which is a negative regulator of the ERK/MAPK signal transduction pathway involved in palatal shelf elevation, resulted in cleft palate via FGF signaling [103], indicating that over-activation of ERK/MAPK can cause cleft palate.

### 5.2. FGFR2-Related Signaling Network in Epithelial–Mesenchymal Interactions during Palatogenesis

In addition to the FGF signaling pathway, the sonic hedgehog (SHH), bone morphogenetic protein (BMP)/transforming growth factor-β (TGF-β), and wingless-type MMTV integration site (WNT) signaling pathways are crucial to palatogenesis. These pathways interact as a complex network regulating development of the palate. Furthermore, the developing palate consists of multiple cell types, including the neural crest-derived palatal mesenchyme, the ectoderm-derived epithelium, of which the most apical layer is composed of periderm cells, and the cranial paraxial mesoderm-derived myogenic cells in the soft palate [30]. Reciprocal epithelial–mesenchymal interactions control the growth and patterning of the palatal shelf [28].

SHH is a morphogen that plays a crucial role in organizing the development of the anterior secondary palate [104,105]. It is expressed in the rugal stripes on the oral side of the developing hard palate. This pattern is established by a Turing mechanism, with FGF and SHH as components of an activator–inhibitor pair in this system [106]. *Shh* is a downstream target of FGF10/FGFR2b signaling [104]. The mesenchymally expressed FGF10 regulates the expression of SHH in the palatal epithelium, which in turn signals back to the mesenchyme to control anterior palatal growth and patterning [104,105].

**Figure 3:**
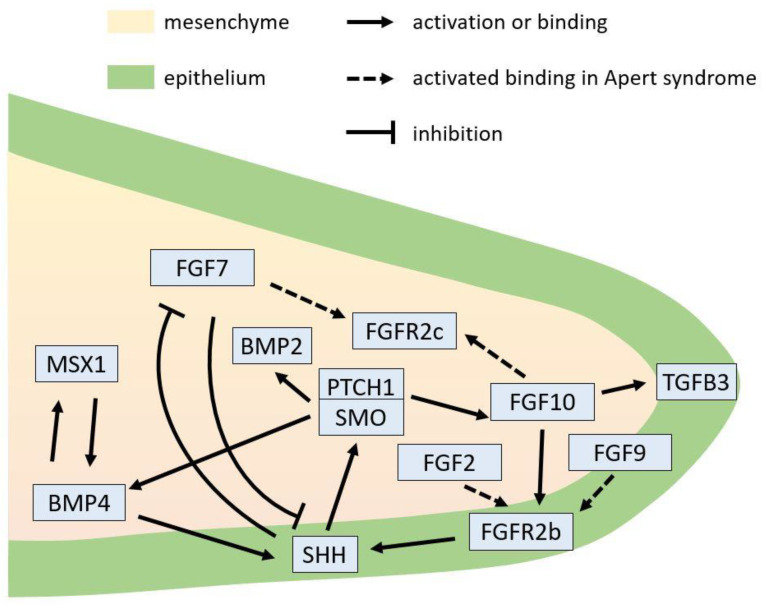
Potential signaling regulations involved in palatal defects in Apert syndrome during palatal shelf growth.

FGF10 is a crucial mesenchymal signal required for palatal outgrowth. FGF10 contributes to the skeletal and visceral defects of the Apert syndrome mouse model created by deletion of *Fgfr2* exon IIIc [107]. The expression of *Fgf10* was reduced in the palatal mesenchyme of embryos lacking mesenchymal expression of the HH receptor, Smoothened (*Smo*), indicating that SHH and FGF10 function in a positive-feedback loop [28,105]. Deletion of *Shh* in the palatal epithelium (by *K14*-Cre) or of *Smo* in the palatal mesenchyme (by *Osr2*-Cre) results in cleft palate [105]. In addition, knockout of *Fgf10* or *Fgfr2b* also causes cleft palate [104], suggesting the important role of the FGF10–FGFR2b–SHH regulatory loop in palate development (Figure 3). Epithelial-specific deletion of *Fgfr2* in *K14*-Cre; *Fgfr2^fl/fl^* mice results in cleft palate, indicating that FGFR2 in the epithelium is crucial for palate development [108].

FGF7 is expressed mainly in the nasal region of the palatal mesenchyme before elevation. In contrast, FGF10 is expressed preferentially on the oral side [104,109]. In palatal explant cultures, FGF10 induced *Shh* expression in the palatal epithelium, whereas FGF7 repressed its expression [104,109]. In addition, SHH protein inhibited *Fgf7* expression in palatal mesenchyme explants [109]. Therefore, SHH signaling regulates the oronasal asymmetry of the palatal shelves through differential regulation of expression of *Fgf10* and *Fgf7* in the mesenchyme [26,109].

SHH signaling also regulates *Bmp2* and *Bmp4* expression in the developing palatal mesenchyme [105,110]. In the anterior palatal shelves, mesenchymally expressed MSX1, which can be induced by BMP4, is required for BMP4 expression in the palatal mesenchyme. BMP4 maintains *Shh* expression in the MEE and SHH in turn induces *Bmp2* expression in the mesenchyme. BMP2 functions to induce cell proliferation in the palatal mesenchyme and promote palatal growth [110].

*Tgfb3* is expressed in palatal epithelium and is regulated by FGF10 during palatogenesis [111]. Britto et al. analyzed the expression of proteins in FGF signaling and TGFβ3 throughout the temporospatial sequence of human palatal shelf fusion [112]. The coexpression of TGFβ3 with FGFs and FGFRs suggests that human palatal fusion in the midline and at the nasal septal junction is coregulated by FGF and TGFβ3 signaling [112]. The role of TGF-β in the palatal defects of Apert syndrome is still unknown.

The potential signaling regulations in the palatal shelf involved in palatal defects in Apert syndrome are illustrated in Figure 3.

## 6. Open Questions and Future Directions

In patients with Apert syndrome, high-arched palate with lateral palatal swelling is the most common palatal anomaly (70–100%, Table 1). Approximately 50% of patients presented with cleft palate (Table 1 and Table 3), and 30% of patients presented with bifid uvula [37,113]. The incidence of cleft palate and other palatal defects in patients with Apert syndrome varies between different studies. This may be caused by factors such as genetic background, environmental effects, variability of phenotypes, and diagnostic criteria across studies.

Craniosynostosis is a diagnostic feature of Apert syndrome. Almost all Apert patients have coronal craniosynostosis, and a majority have sagittal and lambdoid craniosynostosis [8,39]. A subset of affected individuals have cleft palate. A wide array of other abnormalities is seen in Apert syndrome patients, including craniofacial malformations, syndactyly, feeding problems, cognitive disorders, hearing loss, and speech and language difficulties. Thus, a multidisciplinary team is essential to provide treatment and care. For cleft palate, various treatments including surgery, dental management, speech therapy, and psychological support are required [2]. Palate repair surgery is typically performed prior to development of pressure consonants to improve speech production and intelligibility [8]. However, patients may experience lifelong psychosocial effects from the malformation of the facial appearance even after surgeries [2]. Future studies can be performed to determine if there is a correlation between the palatal phenotype and other phenotypes and management issues in Apert syndrome.

Various potential therapies are emerging to remedy cleft palate. One potential therapeutic avenue applicable to Apert syndrome is targeting of FGFR activity and downstream signaling pathways. For example, coronal suture fusion in calvarial explants was decreased by exposure to the MEK1 inhibitor PD98059 in the FGFR2 P253R model [86]. Both pre- and postnatal administrations of the MEK1/2 inhibitor U0126 alleviated symptoms in the FGFR2 S252W model, as did a gene therapy strategy of expressing a short hairpin RNA against the *Fgfr2^S252W^* allele [114]. FGFR2-related signaling network analysis may help to find targets for novel drugs to treat or alleviate symptoms. In addition, innovative cellular therapeutics such as stem cell transplantation have been applied in cleft palate treatment [115,116]. Mazzetti et al. reported results from patients with cleft lip and palate who had stem cells from umbilical cord blood and placental blood injected into the bone and soft tissue during the primary surgical repair procedure. Compared to controls, the group with stem cell injection showed improvement in the inflammatory response, fewer postoperative complications and less fibrosis. Tomography showed an improved maxillary alignment, and the alveolar cleft became smaller [116].

High-arched palate has been reported in ciliopathy-related syndromes [117,118]. Ciliopathies are disorders that arise from the dysfunction of motile and/or non-motile cilia [119], with craniofacial dysmorphology as a common feature [118]. An etiological link between ciliopathies and FGF hyperactivation syndromes has been identified [118]. The primary cilium is the central organelle for the transduction of the hedgehog signaling pathway in vertebrates and is also a signaling center for other signaling pathways, such as WNT, Notch, Hippo, GPCR, PDGF, and other RTKs including FGF, mTOR, and TGF-β [120,121]. Considering that FGF signaling regulates the formation of primary cilia [121], it would be interesting to investigate the roles of cilia and associated signaling pathways in the palatal defects in Apert syndrome, especially the high-arched palate.

## 7. Conclusions

Apert syndrome is a rare genetic disorder caused by pathogenic variants of the *FGFR2* gene. Cleft palate is a common phenotype in Apert syndrome cases, and high-arched palate, lateral palatal swelling, and bifid uvula also occur with high frequency. Palatal defects in Apert syndrome make evident the critical role of FGFR2 in palatogenesis. Mouse models of Apert syndrome have been established and display many phenotypes of Apert syndrome. In mouse models of FGFR2 S252W and FGFR2 P253R, incomplete closure of the anterior end of the secondary palate occurs in newborn mice. These models provide opportunities for in vivo investigation of the role of FGF signaling in palatal defects in Apert syndrome. The pathogenic variants in FGFR2 potentially alter a complex signaling network in epithelial–mesenchymal interactions during palatogenesis resulting in cleft palate.

## Figures and Tables

**Table 1:** Palatal phenotypes in cohort studies of Apert syndrome.

Study	Palatal Phenotypes	References
Solomon et al. (1973)	In a cohort of 13 patients, all 13 (100%) presented with a Byzantine palatal arch; 6 of 13 (46%) presented with a bifid uvula; 3 of 13 (23%) presented with a cleft of the soft palate.	[36]
Peterson; Pruzansky (1974)	In a cohort of 19 patients, all 19 (100%) presented with a narrow, high-arched palate with lateral accumulations of soft tissue barely separated by a deep median groove; 6 of 19 (32%) presented with a bifid uvula; 2 of 19 (11%) presented with a cleft palate. On radiographic examination, 10 of 19 (53%) presented with abnormal length of the soft palate, and 8 of 19 (42%) showed abnormal velar thickness (4 overlapping cases).	[37]
Peterson-Falzone et al. (1981)	In a cohort of 29 patients, alterations of the nasopharyngeal architecture were found. Hard palate length was reduced and soft palate length was greater than the norm.	[38]
Kreiborg; Cohen (1992)	In a cohort of 119 patients, almost all patients presented with a Byzantine arch-shaped palate; approximately 75% of patients presented with a cleft of the soft palate or bifid uvula.	[32]
Cohen; Kreiborg (1996)	In a cohort of 136 patients ^1^, almost all patients (94%) presented with a highly arched, constricted palate and median furrow. Lateral palatal swellings were present that increased in size with age. The hard palate was shorter than normal and the soft palate was both longer and thicker than normal.	[39]
Arroyo Carrera et al. (1999)	In a cohort of 17 patients, 4 of 17 (23.5%) presented with a cleft palate.	[40]
Albuquerque; Cavalcanti (2004)	In a cohort of 5 patients, all (100%) presented with a pseudo-cleft in the midline palate.	[41]
Letra et al. (2007)	In a cohort of 23 patients, 16 of 23 (70%) presented with an arched palate; 21 of 23 (91%) presented with lateral gingival swellings; 1 of 23 (4%) presented with a cleft of the soft palate.	[35]
Stavropoulos et al. (2012)	In a cohort of 23 patients with Apert syndrome and 28 patients with Crouzon syndrome, cleft palate and extensive lateral palatal soft tissue swellings were more common in children with Apert syndrome than Crouzon syndrome.	[42]
Kakutani et al. (2017)	In a cohort of 5 patients, all 5 (100%) had a pseudo-cleft palate with a Byzantine arch shape; 4 of 5 (80%) presented with narrowing in the upper arch.	[43]
Kobayashi et al. (2021)	In a cohort of 7 patients, all 7 (100%) had a high palate with lateral palatal swellings; 2 of 7 (28.6%) presented with a cleft of the soft palate; 1 of 7 (14.3%) presented with a cleft of the hard palate.	[33]
Ogura et al. (2022)	In a cohort of 4 patients, 2 of 4 (50%) presented with a cleft of the soft palate; 1 of 4 (25%) presented with a cleft of the hard palate.	[44]

^1^ An enlarged cohort from the study with 119 samples by Kreiborg; Cohen (1992) [32].

**Table 2:** Palatal phenotypes in case reports of Apert syndrome.

Study	Palatal Phenotypes	References
Batra et al. (2002)	A female patient had a pseudo-cleft palate.	[45]
Vijayalakshmi; Menon (2002)	A male patient had a cleft of the soft palate.	[46]
Huang et al. (2004)	A female patient had a submucous cleft palate and absent uvula.	[47]
Verma et al. (2005)	A male patient had a cleft palate.	[48]
Tosun et al. (2006)	A male patient had a V-shaped maxillary arch with a midline pseudo-cleft and lateral swellings on the palatal process.	[49]
Herman; Siegel (2010)	A female patient with an S252W variant in the *FGFR2* gene had a cleft of the soft palate.	[50]
Premalatha et al. (2010)	A male patient had a high-arched palate with a pseudo-cleft in the posterior one-third.	[51]
Şoancǎ et al. (2010)	A male patient had a Byzantine arch palate associated with lateral swellings of the palatine processes and a bifid uvula.	[52]
Vadiati Saberi; Shakoorpour (2011)	A female patient had an arched swelling (pseudo-cleft configuration) and a V-shaped maxillary arch.	[53]
Costa et al. (2012)	A female patient had a U-shaped dental arch, swelling of the lateral palatine processes on both sides, and a bifid uvula.	[54]
Ileri; Goyenc (2012)	A female patient had a cleft palate.	[55]
Khan et al. (2012)	A male patient had a V-shaped maxillary arch and a pseudo-cleft palate.	[56]
Bhatia et al. (2013)	A male patient had a deep pseudo-cleft.	[57]
Aggarwal et al. (2014)	A male patient had a bulky, high-arched V-shaped palate with an occult submucosal cleft and rotated maxillary and mandibular incisors.	[58]
Ercoli et al. (2014)	A male patient had a high-arched palate.	[59]
Kumar et al. (2014)	A male patient had a high-arched palate associated with lateral swellings of the palatine processes on either side of the midline, mimicking a pseudo-cleft.	[60]
Spruijt et al. (2015)	A male patient with an S252W variant in the *FGFR2* gene had a high-arched, narrow palate.	[61]
Barman et al. (2015)	A male patient had a high-arched palate.	[62]
Torres et al. (2015)	A male patient had a high-arched palate. A variant NM_000141.5: c.939+42T>A (T78.501A) located near the splicing site in *FGFR2* was found.	[63]
Işık et al. (2017)	A female patient had a cleft palate.	[64]
Cha et al. (2018)	A male patient had a narrow and triangular-shaped maxillary arch and Byzantine arch-shaped palate.	[65]
Barro et al. (2019)	A female patient had a cleft palate.	[66]
Brajadenta et al. (2019)	An Indonesian male patient with an S252W variant in the *FGFR2* gene had maxillary hypoplasia with a high-arched palate. His V-shaped maxillary arch was filled with double rows of teeth.	[67]
Cammarata-Scalisi et al. (2019)	One of two unrelated female patients, one had a high-arched palate, and the other a cleft of the soft palate. In both patients, a heterozygous S252W variant was identified.	[68]
Dap et al. (2019)	One of two monozygotic twins with an S252W variant was found to have a cleft palate at 30 weeks of gestation.	[69]
Kumar et al. (2019)	A female patient had a high-arched palate, a pseudo-cleft, and gingival enlargement.	[70]
Chavda et al. (2021)	A male patient had a high-arched palate.	[71]
Jose et al. (2021)	A female patient had a pseudo-cleft.	[72]
Tonni et al. (2022)	A female fetus at 20 weeks of gestation was found to have a smooth palate with a midline cleft and an absent uvula. A heterozygous P253R variant was identified.	[73]

**Table 3:** Genotype–phenotype correlations of cleft palate with the FGFR2 S252W and P253R variants.

Study	Cohort			FGFR2 S252W		FGFR2 P253R		Notes	References
	Cohort Size	Abnormal Palate Number	%	Cleft Palate Fraction	Cleft Palate %	Cleft Palate Fraction	Cleft Palate %		
Park et al. (1995)	36	Cleft palate in 16 patients	44.4	15/16	93.8	1/16	6.2		[16]
Slaney et al. (1996)	87	Soft palate cleft or bifid uvula in 37 patients	42.5	24/41	58.5	4/23	17.4		[78]
Lajeunie et al. (1999)	36	Cleft palate in 15 patients	41.7	12/23	52.2	2/12	16.7	One fetus with the S252F mutation also had a cleft palate	[17]
Sakai et al. (2001)	6	Cleft palate in 5 patients	83.3	5/5	100.0	0/1	0.0		[81]
Von Gernet et al. (2000)	21	Cleft palate in 11 patients	52.4	9/15	60.0	2/6	33.3		[79]
Kilcoyne et al. (2022)	51	Cleft palate or bifid uvula in 26 patients.	51.0	18/28	64.3	8/23	34.8		[80]

**Table 4:** Palatal phenotypes in Apert syndrome-related mouse models.

Study	Model	Palatal Phenotypes	References
Wang et al. (2005)	* Fgfr2^+/S252W^*	Malformation of the palate. The structures of the face and palate were 40–50% smaller in the mutant mouse than in the control mouse. Incomplete closure of the anterior end of the secondary palate at P1.	[84]
Wang et al. (2010)	* Fgfr2^+/P253R^*	From E15.5 until P0, all *Fgfr2^+/P253R^* exhibited bilateral incomplete fusion at the junction of the primary and secondary palatal shelves. The developing mutant palates were shorter.	[85]
Martínez-Abadías et al. (2013)	*Fgfr2^+/S252W^* and *Fgfr2^+/P253R^*	Contracted and separated palatal shelves, and premature fusion of the maxillary–palatine suture in *Fgfr2^+/S252W^* and *Fgfr2^+/P253R^* mice. *Fgfr2^+/S252W^* mice display relatively more severe palatal dysmorphology.	[91]
Holmes; Basilico (2012)	* EIIA-Cre;Fgfr2^NeoS252W/+^*	Incomplete closure of the anterior end of the secondary palate at P0.	[90]
	*Wnt1-Cre;Fgfr2^NeoS252W/+^*	Complete palatal closure at P0.	[90]
	*Mesp1-Cre;Fgfr2^NeoS252W/+^*	Complete palatal closure at P0.	[90]

## Data Availability

Not applicable.
